# Design of double-notch UWB filter with upper stopband characteristics based on ACPW-DGS

**DOI:** 10.1371/journal.pone.0282060

**Published:** 2023-02-22

**Authors:** Mingming Gao, Xueman Zhang, Xitao Chen, Jingchang Nan

**Affiliations:** 1 College of Electronics and Information Engineering, Liaoning Technicial University, Huludao, Liaoning Province, China; 2 College of Information Science and Technology, Dalian Maritime University, Dalian, Liaoning Province, China; 3 CCTEG shenyang research institute, Fushun, Liaoning Province, China; Edinburgh Napier University, UNITED KINGDOM

## Abstract

In this manuscript, a compact (size only 9.8mm*9.8mm) Ultra Wide Band (UWB) bandpass filter with a new structure is proposed, which can be used in the UWB wireless communication band authorized by the FCC. The top plane is composed of a pair of back-to-back microstrip lines, and the ground plane structure is based on an asymmetric coplanar waveguide-defect ground structure (ACPW-DGS). UWB is formed by the vertical electromagnetic coupling of the top plane and the ground plane. On this basis, split ring resonator (SRR) and C type resonator (CTR) are utilized to place double notch bands. A novel third order nested C-type resonator (TONCTR) is obtained by performing CTR, which can further optimize the upper stopband while ensuring double notch bands. The filter can be used for filtering within the UWB system, and it can also avoid the amateur radio band (9.2 -10.3GHz) and the X-band satellite link band (9.6-12.3GHz) on UWB communication systems. Finally, the measured results from the fabricated prototype are basically consistent with the simulation results.

## 1 Introduction

Since the United States Federal Communication Commission (FCC) used the unlicensed UWB for short-distance transmission in 2002 [1], UWB research has attracted extensive attention from scholars around the world. Especially in the global outbreak of COVID-19 in recent years [2], in order to suppress the spread of the virus, people are working from home. The simultaneous login of a large number of users is a very big challenge compared with traditional communication systems, and bandwidth resources are very limited. At this time, the advantages of the UWB communication system are fully reflected. It not only has a wider bandwidth, but also has a high data rate within a short-distance transmission range [3], which can satisfy a large number of users to surf the Internet at the same time.The filter is an important part of the radio frequency (RF) transmission system, the combination of UWB and filter design has become a current research hotspot. The design of UWB filters mainly includes the following methods [4–13]: high-pass/low-pass cascade [5], loading short-circuit stub [6–8], multi-mode resonator method [9, 10], hybrid microstrip/CPW method [11–13] and so on. The CPW design perfectly meets the requirements of miniaturization of microwave RF devices under the background of 5G wireless communication. At the same time, in order to avoid the interference of the bands which divided finely in the modern communication system to the UWB system, the loaded resonator method [14] and the defect microstrip structure method [15] are used to design UWB filters with notch characteristics.

Reference [5] achieves a UWB response by cascading a low-pass filter (LPF) with a defect structure and a short-circuit stepped impedance high-pass filter (HPF). The narrow notch band is obtained by embedding two stepped impedance stubs, and its depth reaches -60dB. The number of notches is small, so it’s difficult to avoid interference caused by other bands in the UWB system at the same time. Reference [16] proposed a new three-mode step impedance resonator (TSIR), the TSIR structure consists of two T-type SIRs and a 1/4 wavelength uniform impedance resonator (UIR), these notch bands are controlled by three parts separately. Although the number of notches is large, the depth of the notches is not enough, only reaching -15dB. In [17], the hybrid microstrip/CPW method was used to achieve UWB, a new sectoral folded split ring resonator (SFSRR) and a spiral resonator (SR) are used to generate double notch bands, and its design size is miniaturized. However, the design does not consider the stop-band characteristics, and the out-of-band suppression capability is poor, only reaching 16GHz. Reference [18] proposed symmetric and asymmetric plasmonic bandpass filter (BPF) topologies based on the metal–insulator–metal (MIM) configuration, it is observed that the resonance mode of proposed BPFs can be tuned by changing the nanodisk resonator radius. They can be easily tuned for other desired wavelengths. A compact plasmonic bandpass filter based on metal-insulator-metal plasmonic waveguides and a coupled U-shaped cavity is proposed in reference [19]. By tuning the height of the designed cavity, it is found that the resonance wavelength can be easily adjusted. Furthermore, the quality factor of the designed filter can be increased by increasing the cavity numbers.

This manuscript overcomes the shortcomings of the above references, and proposes a dual-notch UWB filter with good high stop-band characteristics. Firstly, the top plane structure is composed of a pair of back-to-back microstrip lines symmetrical with a 45° axis, any of which is composed of an input/output microstrip line and a vertical microstrip line connecting two arcs with different tangent angles, the ground plane consists of ACPW and DGS. The above two form UWB through vertical electromagnetic coupling. Secondly, two notches are generated through SRR and CTR. Finally, optimize the second notch structure and propose TONCTR. The filter uses a Rogers RT/duroid6006 dielectric substrate with a relative dielectric constant of 6.15, its loss tangent is 0.0019, the thickness of the substrate is 0.635 mm, and the size is only 9.8 mm × 9.8 mm. The three-dimensional electromagnetic simulation software HFSS 15.0 is used for simulation optimization, and the ADS software is used to design the equivalent circuit. And the test results from the fabricated prototype are basically consistent with the simulation results.

## 2 UWB BPF design

**Fig 1:**
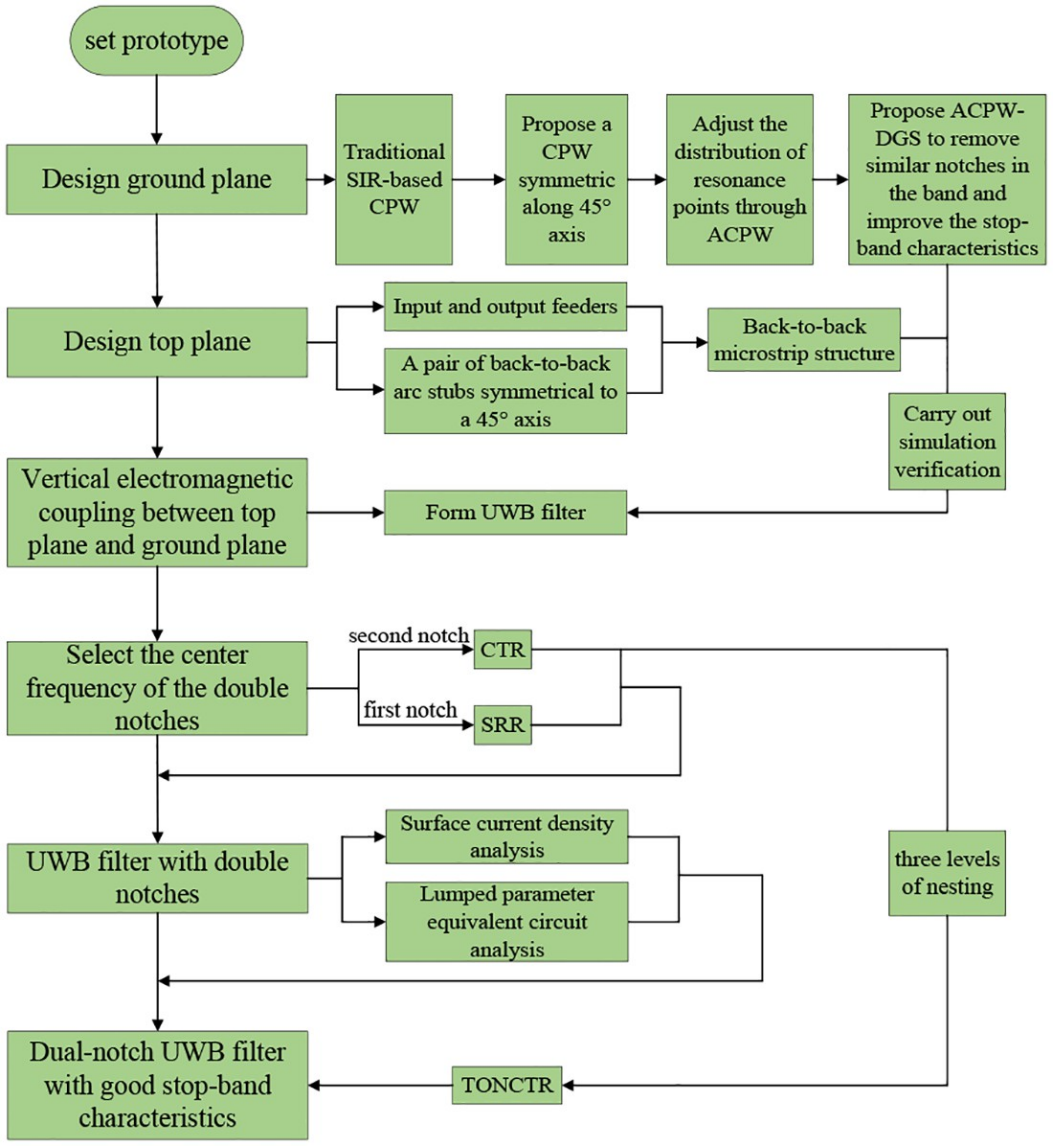
Design flow chart.

The UWB filter design flow chart in this manuscript is shown in Fig 1. The realization of the UWB filter is formed by the vertical electromagnetic coupling of the top plane (back-to-back microstrip line structure) and the ground plane (ACPW-DGS), double notches are introduced through the SRR and CTR structures respectively, and the CTR is nested in three layers to form TONCTR, so as to obtain good stop-band characteristics.

### 2.1 CPW design

**Fig 2:**
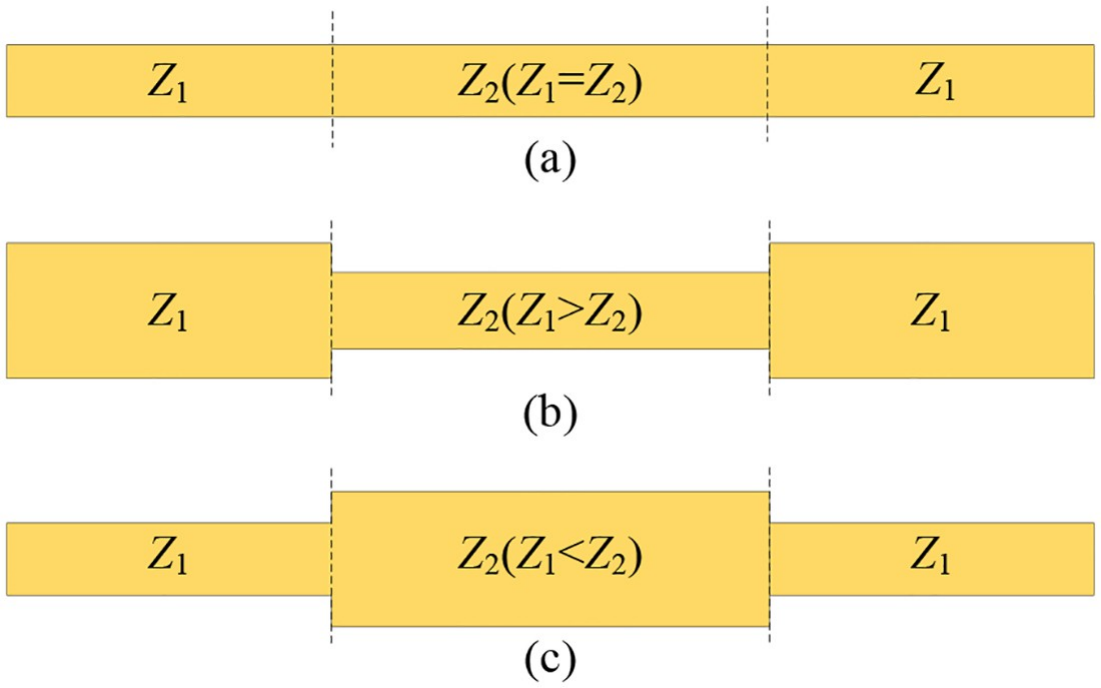
Half-wavelength open transmission line resonator with impedance ratio *K*. (a) *K* = 1; (b) *K* <1; (c) *K* >1.

According to the impedance ratio of adjacent transmission lines *K* = *Z*_2_/*Z*_1_, the transmission lines can be subdivided into three types [20]: UIR (Uniform Impedance Resonator, *K* = 1), SIR_1_ (Stepped Impedance Resonator, *K* <1), SIR_2_ (*K*>1), as shown in Fig 2(a)–2(c) respectively.

The resonance occurrence condition of a half-wavelength resonator with uniform linewidth is shown in Eq (1):

$$\begin{array}{c} \theta_{T}=\theta_{2}+2\theta_{1}+\theta_{2}=n\pi \end{array}$$ (1)

where *θ*_T_ is the electrical length of the entire resonant unit, and n refers to the number of resonant modes.

From the above equation, we can get that the resonant frequency of the high-order mode is always several times the resonant frequency of the fundamental mode, which is not conducive to the design of single-channel filters. In order to solve this problem, a common method is to change the uniform line width to a non-uniform mode. So far, the importance of SIR is self-evident. Compared with UIR, the biggest advantage of SIR is that SIR can increase the frequency ratio of the first few high-order modes to the fundamental mode, so that it can be adjusted to a suitable position and arranged as evenly as possible in the passband. It is particularly important in the design of ultra-wideband filters. Therefore, SIR is widely used when designing filters with band-pass performance [21].

**Fig 3:**
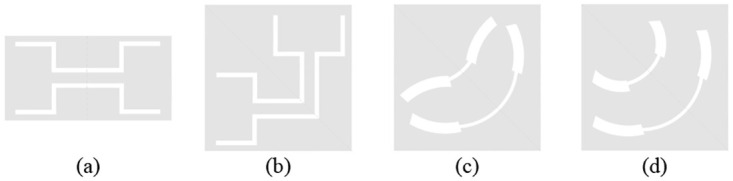
The evolution process of CPW. (a) traditional SIR-based CPW. (b) symmetry to 45° axis. (c) arc type. (d) uniform impedance change.

**Fig 4:**
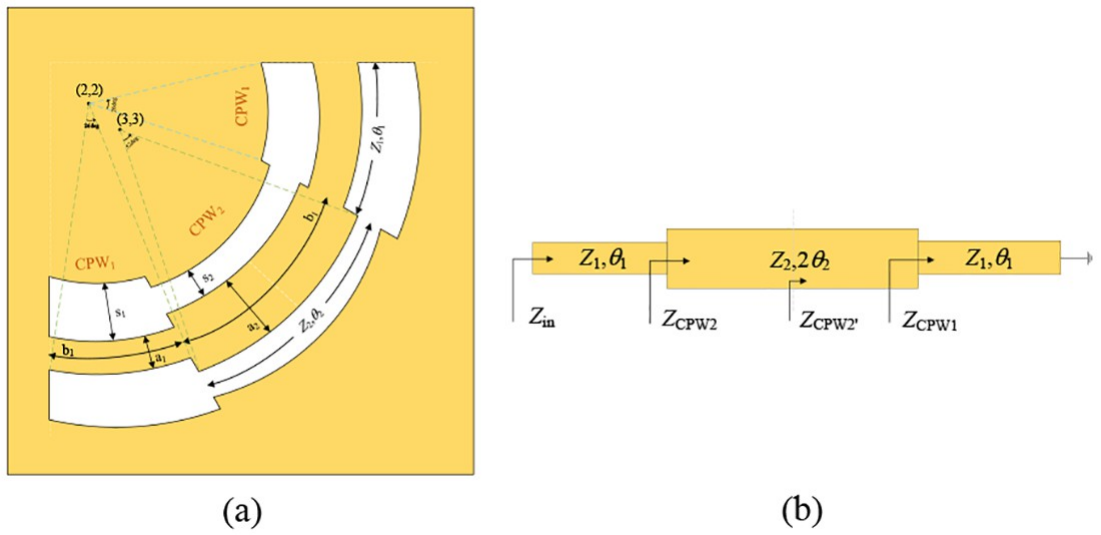
CPW equivalent analysis. (a) arc-shaped coplanar waveguide. (b) traditional SIR.

Fig 3 shows the evolution process of the CPW design in this manuscript: Fig 3(a) is a traditional SIR-based CPW, the input and output ports are optimized from left-right symmetry to 45° axis, as shown in Fig 3(b); in order to reduce the influence of the impedance caused by the right angle, the arc type is used instead of the right angle, and the evolution structure is shown in Fig 3(c). Aimed at meeting the uniform impedance change of the central transmission conductor strip, the optimized structure is shown in Fig 3(d). The arc-shaped coplanar waveguide can be equivalent to a traditional SIR for analysis, as shown in Fig 4(a) and 4(b) respectively. The input impedance expression at a distance *l* from the load on a uniform lossless transmission line is Eq (2):

$$\begin{array}{c} Z_{\text{in}}=Z_{0}\frac{Z_{L}+jZ_{0}\text{tan}\left(\beta l\right)}{Z_{0}+jZ_{L}\text{tan}\left(\beta l\right)} \end{array}$$ (2)

where *β* *l* = *θ*. For the analysis of coplanar waveguides, it can be equivalent to a stepped impedance resonator with short-circuit terminals. Looking from left to right, *Z*_CPW1_ can be regarded as the input impedance of the terminal short circuit, and $Z_{\text{CPW}2^{\prime }}$, *Z*_CPW2_, $Z_{\text{in}}$ can be regarded as the input impedance of the terminal which connection pure reactance. The expressions of *Z*_CPW1_, $Z_{\text{CPW}2\prime }$, *Z*_CPW2_, and *Z*_in_ can be represented by equations Eqs (3)–(6), respectively:

$$\begin{array}{c} Z_{\text{CPW}1}=jZ_{1}\text{tan}\theta_{1} \end{array}$$ (3)

$$\begin{array}{c} Z_{\text{CPW}2^{\prime }}=Z_{2}\frac{Z_{\text{CPW}1}+jZ_{2}\text{tan}\theta_{2}}{Z_{2}+jZ_{\text{CPW}1}\text{tan}\theta_{2}}=jZ_{2}\frac{\text{tan}\theta_{1}+K\text{tan}\theta_{2}}{K-\text{tan}\theta_{1}\text{tan}\theta_{2}} \end{array}$$ (4)

$$\begin{array}{c} Z_{\text{CPW}2}=Z_{2}\frac{Z_{\text{CPW}2^{\prime }}+jZ_{2}\text{tan}\theta_{2}}{Z_{2}+j{}_{\text{CPW}2^{\prime }}\text{tan}\theta_{2}}=jZ_{2}\frac{\text{tan}\theta_{1}+2K\text{tan}\theta_{2}-\text{tan}\theta_{1}tan^{2}\theta_{2}}{K-2\text{tan}\theta_{1}\text{tan}\theta_{2}-K{\text{tan}}^{2}\theta_{2}} \end{array}$$ (5)

$$\begin{array}{c} Z_{\text{in}}=jZ_{1}\frac{2\left(K\text{tan}\theta_{1}+\text{tan}\theta_{2}\right)\left(K-\text{tan}\theta_{1}\text{tan}\theta_{2}\right)}{K\left(1-{\text{tan}}^{2}\theta_{1}\right)\left(1-{\text{tan}}^{2}\theta {}_{2}\right)-2\left(1+K^{2}\right)\text{tan}\theta_{1}\text{tan}\theta_{2}} \end{array}$$ (6)

Where, *K* = *Z*_2_/*Z*_1_. Under the condition of resonance: the input impedance *Z*_in_ = 0, Eq (7) can be obtained:

$$\begin{array}{c} K-\text{tan}\theta_{1}\text{tan}\theta_{2}=0 \end{array}$$ (7)

Combined with the Eq (1),the following Eq (8) can be obtained according to the simplification of the trigonometric formula [22, 23]:

$$\begin{array}{c} \text{tan}\frac{\theta_{T}}{2}=\frac{1}{1-K}\left(\frac{K}{\text{tan}\theta_{1}}+\text{tan}\theta_{1}\right),K\neq 1 \end{array}$$ (8)

Differentiate *θ*_1_ in Eq (8) to get Eq (9):

$$\begin{array}{c} \frac{1}{1-K}\left({\text{tan}}^{2}\theta_{1}-K\right){\text{sin}}^{2}\theta_{1}=0 \end{array}$$ (9)

can be obtained

$$\begin{array}{c} \theta_{1}={\text{tan}}^{-1}\sqrt{K} \end{array}$$ (10)

When *θ*_1_ = *θ*_2_ = *θ*, the above Eq (6) can be expressed as Eq (11):

$$\begin{array}{c} Z_{\text{in}}=jZ_{1}\frac{2\left(K+1\right)\left(K-{\text{tan}}^{2}\theta \right)\text{tan}\theta }{K-2\left(1+K+K^{2}\right){\text{tan}}^{2}\theta +K{\text{tan}}^{4}\theta } \end{array}$$ (11)

If the frequency of the first resonant mode is set as *f*_*m*1_, and its corresponding electrical length is set as *θ*_*m*1_, then from the resonance condition:

$$\begin{array}{c} \theta \left(f_{1}\right)={\theta_{m}}_{1}={\text{tan}}^{-1}\sqrt{K} \end{array}$$ (12)

Similarly, let the frequency of the nth resonant mode be *f*_*mn*_ (n = 2,3,4,…), and the electrical length of its pair be *θ*_*mn*_ (n = 2,3, 4,…), according to Eq (11)):

$$\begin{array}{c} \left\{ \begin{array}{c} \text{tan}{\theta_{m}}_{2}=\infty \\ {\text{tan}}^{2}{\theta_{m}}_{3}-K=0\\ \text{tan}{\theta_{m}}_{4}=0 \end{array}\right. \end{array}$$ (13)

So far, it can be deduced that the resonant frequency relationship in the second and third resonant modes are shown in Eqs (14) and (15):

$$\begin{array}{c} \theta \left(f_{2}\right)={\theta_{m}}_{2}=\frac{\pi }{2} \end{array}$$ (14)

$$\begin{array}{c} \theta \left(f_{3}\right)={\theta_{m}}_{3}=\pi -{\text{tan}}^{-1}\sqrt{K} \end{array}$$ (15)

**Fig 5:**
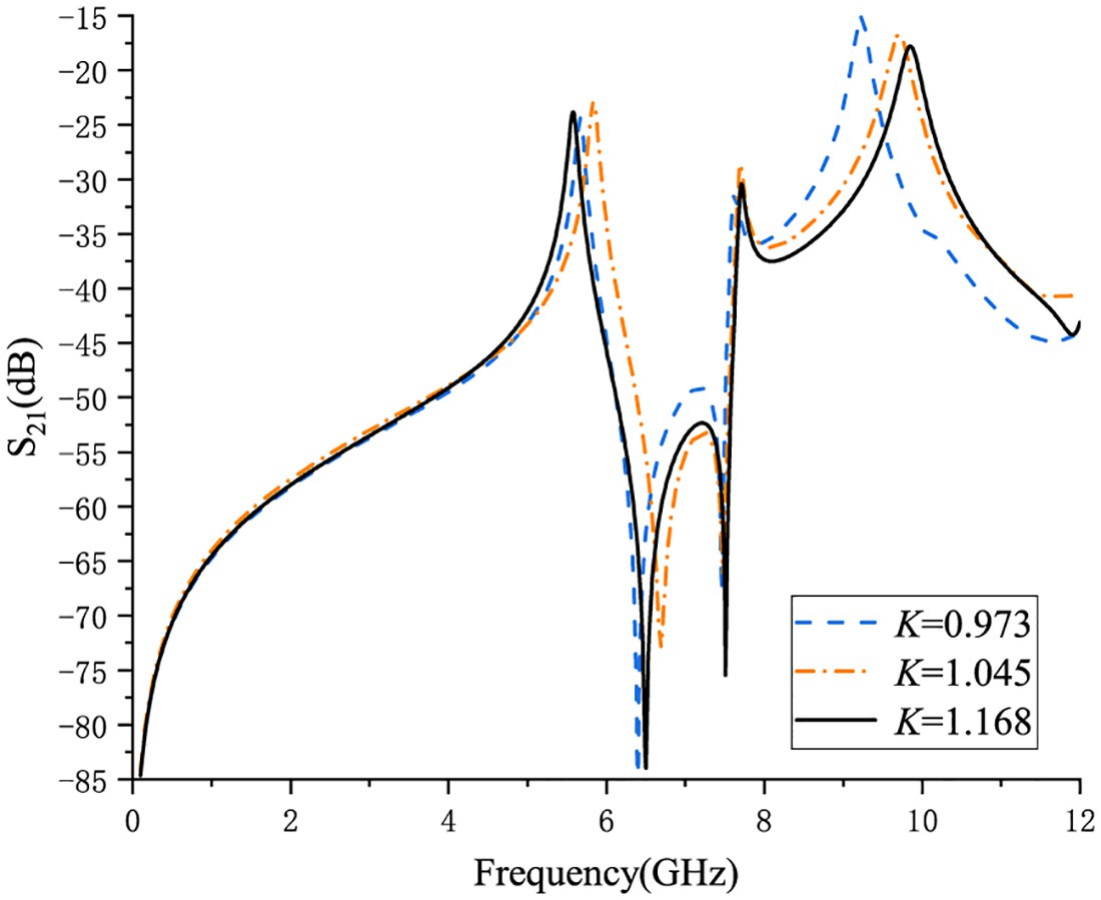
Coupling comparison curves of different *K* values.

According to Eqs (12), (14) and (15), the resonant frequencies *f*_1_, *f*_2_, and *f*_3_ of the first three resonance modes can be obtained. By adjusting the ratio of *s*_1_ and *s*_2_(defined in arc-shaped CPW), the ratio of CPW_1_ and CPW_2_ can be further changed to obtain different *K* values. Fig 5 shows the coupling comparison curves under different *K*. It can be seen from Fig 5 that *f*_1_ and *f*_3_ change with the different *K* values, while *f*_2_ is basically unchanged.

The obtained first three resonance frequencies are normalized to obtain as Eqs (16)–(18):

$$\begin{array}{c} \frac{f_{1}}{f_{1}}=1 \end{array}$$ (16)

$$\begin{array}{c} \frac{f_{2}}{f_{1}}=\frac{\pi }{2*{\text{tan}}^{-1}\sqrt{K}} \end{array}$$ (17)

$$\begin{array}{c} \frac{f_{3}}{f_{1}}=\frac{\pi -{\text{tan}}^{-1}\sqrt{K}}{{\text{tan}}^{-1}\sqrt{K}}=\frac{\pi }{{\text{tan}}^{-1}\sqrt{K}}-1 \end{array}$$ (18)

**Fig 6:**
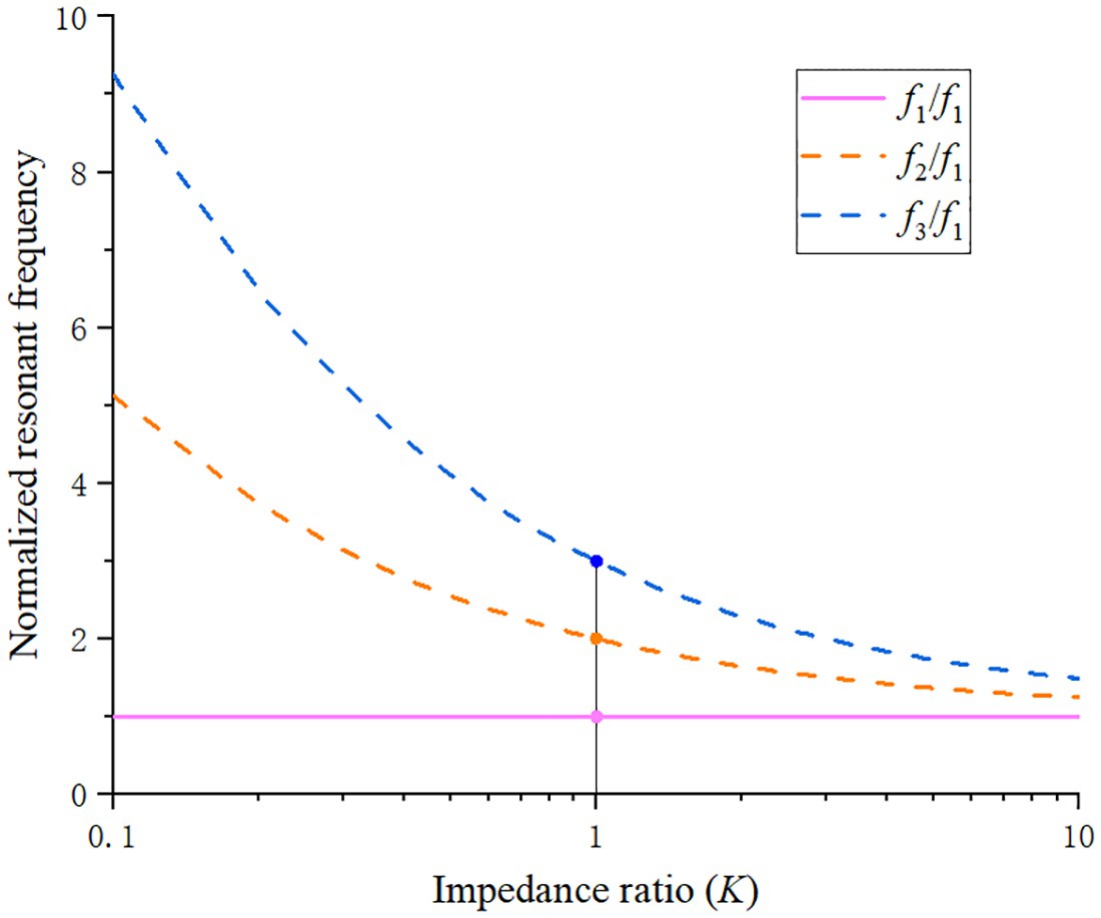
Relationship between normalized resonant frequency and impedance ratio.

Taking *K* (*K* = *Z*_2_/*Z*_1_) as the independent variable, and the above three normalized resonant frequencies as the dependent variable, a curve can be drawn as shown in Fig 6. The impedance ratio *K* ranges from 0.1 to 10. It can be seen from the changing trend of curve in the Fig 6 that the change range of the frequency ratio is obviously different at *K* = 1. When *K* = 1 (*Z*_1_ = *Z*_2_), the resonator is a special uniform impedance; in the range of *K*<1, the ratio of *f*_*x*_ to *f*_1_ increases as the value of *K* decreases, and the resonant mode deviate from the fundamental mode; when *K* >1, the higher-order resonant mode will gradually approach the fundamental frequency. The finally curved SIR structure can be regarded as a combination of three segments of CPW, of which two segments of CPW_1_ are symmetrical along the 45° axis. The impedance and electrical length of CPW_1_ and CPW_2_ can be obtained by calculation, as shown in Table 1. When designing a UWB filter, the ratio should be adjusted to a suitable position, so that the resonant frequency is evenly distributed in the passband, and finally, *K* = *Z*_2_/*Z*_1_ = 0.8 is calculated.

**Table 1:** Corresponding relationship between physical properties and electrical properties of CPW.

	Physical Characteristic			Electrical Characteristics		
CPW_1_	*a*_1_ = 0.616mm	*b*_1_ = 2.014mm	*s*_1_ = 1mm	*Z*_1_ = 97.48Ω	*θ*_1_ = 27.48deg
CPW_2_	*a*_2_ = 0.7mm	*b*_2_ = 4.476mm	*s*_2_ = 0.6mm	*Z*_2_ = 78.24Ω	2*θ*_2_ = 62.83deg

**Fig 7:**
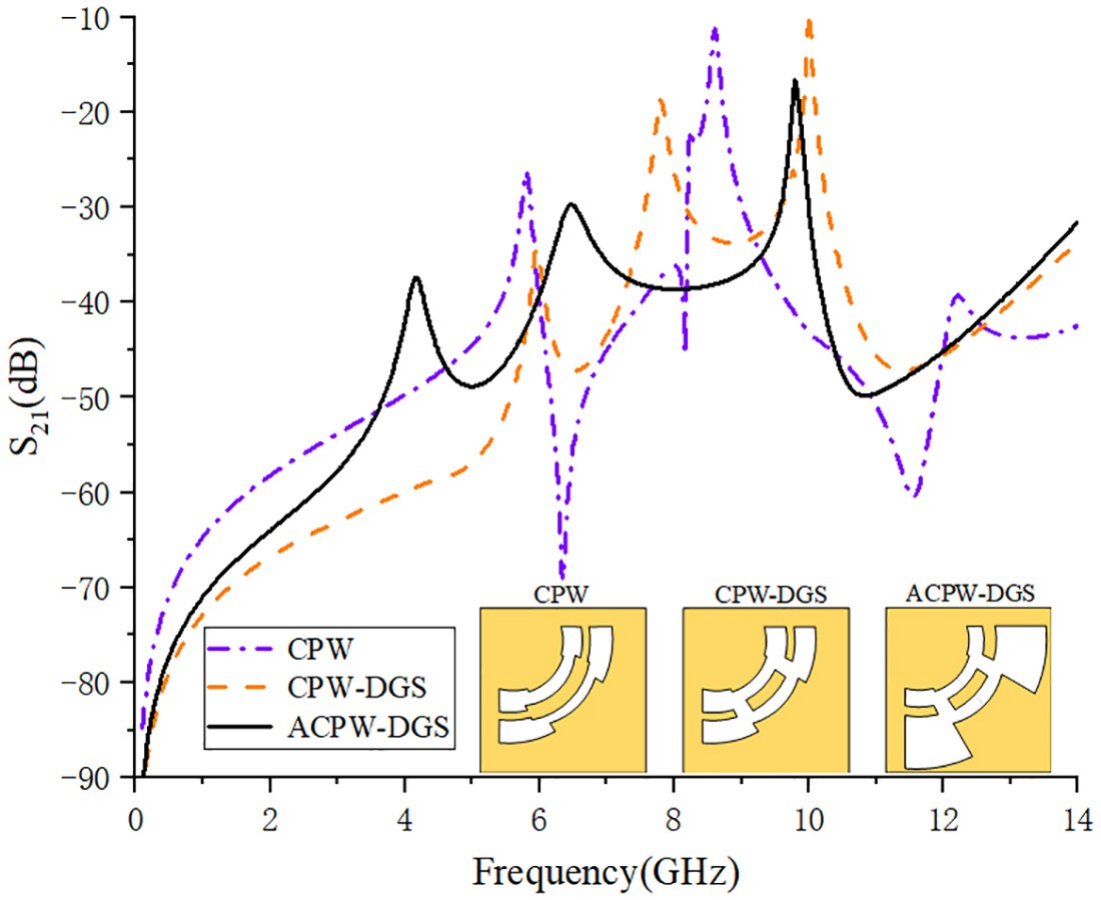
Comparison of weak coupling curves under different structures.

As mentioned above, only the weak coupling curve of the CPW structure has many defects. For example, in the passband range, the resonance point of the weakly coupled curve will cause the passband to collapse under strong coupling. In this manuscript, the DGS structure is adopted on the basis of CPW, which perfectly solves the problem of resonance points in the passband. However, the resonance points under the CPW-DGS structure cannot be perfectly and uniformly distributed in the passband range, so the ACPW-DGS structure is adopted to make the resonance points evenly distributed, as shown in Fig 7.

### 2.2 ACPW design

The coplanar waveguide structure is composed of a central conductor strip and semi-infinite ground planes on both sides. The central conductor strip is used as a signal transmission line, and the strip lines on both sides are used as a grounding line. The interval between the central conductor strip and the grounding line is a slot Line [24–26]. Therefore, the two factors of the width of the central conductor and the width of the slot line become important references for studying the CPW structure. Asymmetric Coplanar Waveguide (ACPW) [27, 28] is a new type of transmission line developed on the basis of traditional CPW, and can also be regarded as one of the deformed structures of CPW. The biggest difference from CPW is that the width of the slot lines on both sides of ACPW is different. Compared with traditional CPW, ACPW has an additional adjustable parameter, so it has more flexibility in the design process of using ACPW. The groove line width *r*_7_ on the ACPW side is studied in detail below.

**Fig 8:**
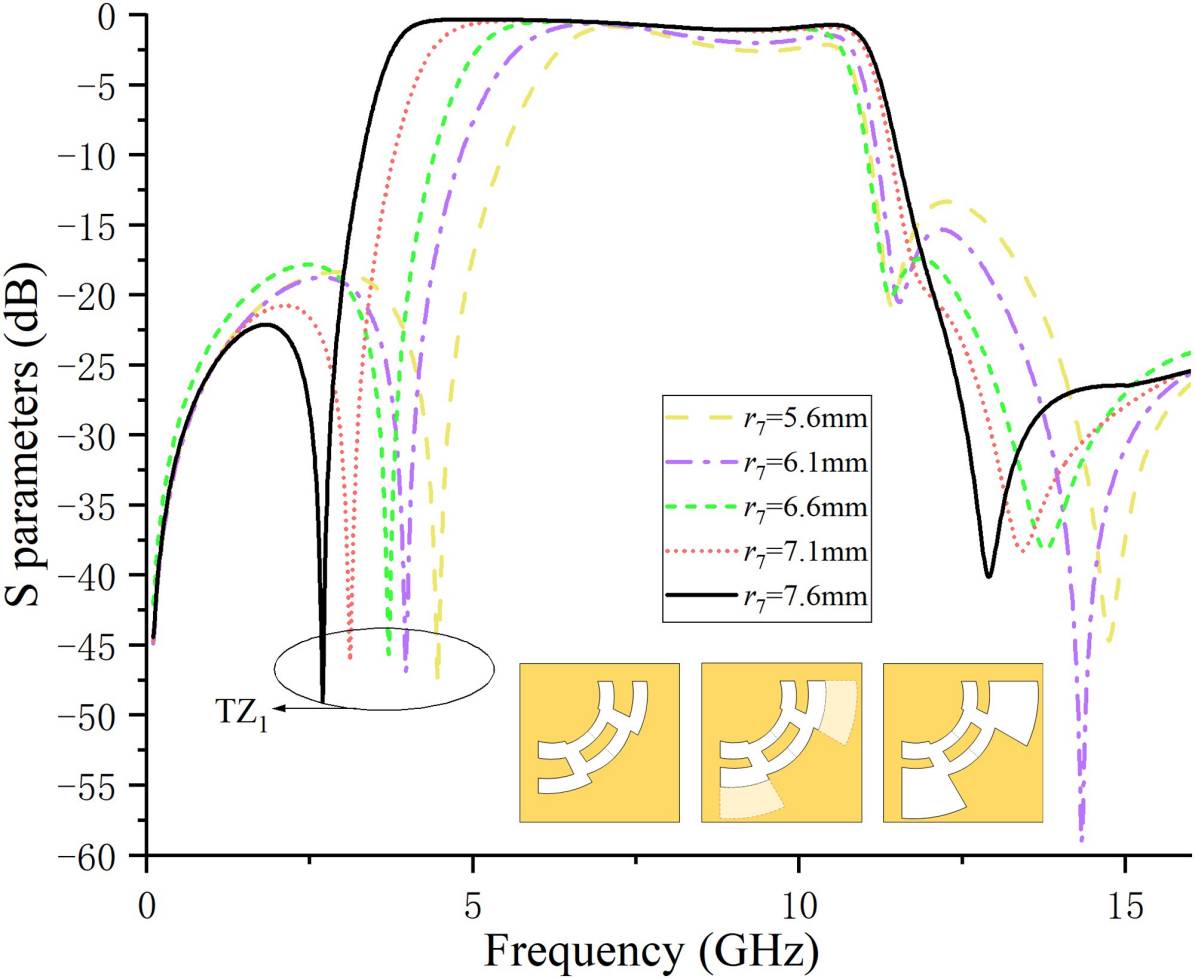
S_21_ waveform when the slot line *r*_7_ keeps increasing.

The slot line widths on both sides of the ACPW central conduction band are different. Therefore, when designing a UWB filter based on ACPW, the slot line width on one side can be fixed, and the performance of the filter can be adjusted by changing the slot line width on the other side.The adjustment method is more conducive to optimizing the filter parameters. In this paper, the upper slot line width is fixed, and the lower slot line width *r*_7_ is modified and tested. The values of *r*_7_ are set to 5.6mm, 6.1mm, 6.6mm, 7.1mm, and 7.6mm respectively, the simulation results are shown in Fig 8. It can be observed from Fig 8 that as the value of *r*_7_ continues to increase, the lower sideband and TZ_1_ continue to move to the low frequency, while the position of the upper sideband is almost unchanged, which is beneficial to the increase of the bandwidth. When the value of *r*_7_ is 7.6mm, the passband has the maximum bandwidth.

### 2.3 ACPW-DGS design

The introduction of defective ground structure (DGS) [29, 30] is an innovation in the design of CPW. In recent years, it is often used to reduce the size of microwave components, this structure changes the current distribution of the transmission line on the ground plane by etching the defect pattern on the ground plane, thereby changing its equivalent capacitance and inductance distribution [31, 32]. The position of the groove line and the width of the groove line are the key parameters in the research. In this paper, the width parameter *α*_5_ of the designed DGS structure is analyzed in detail below. At the same time, it is also a good innovation to add a metal conductor structure to the DGS structure. The design performance of the filter can be adjusted without increasing the design size.

**Fig 9:**
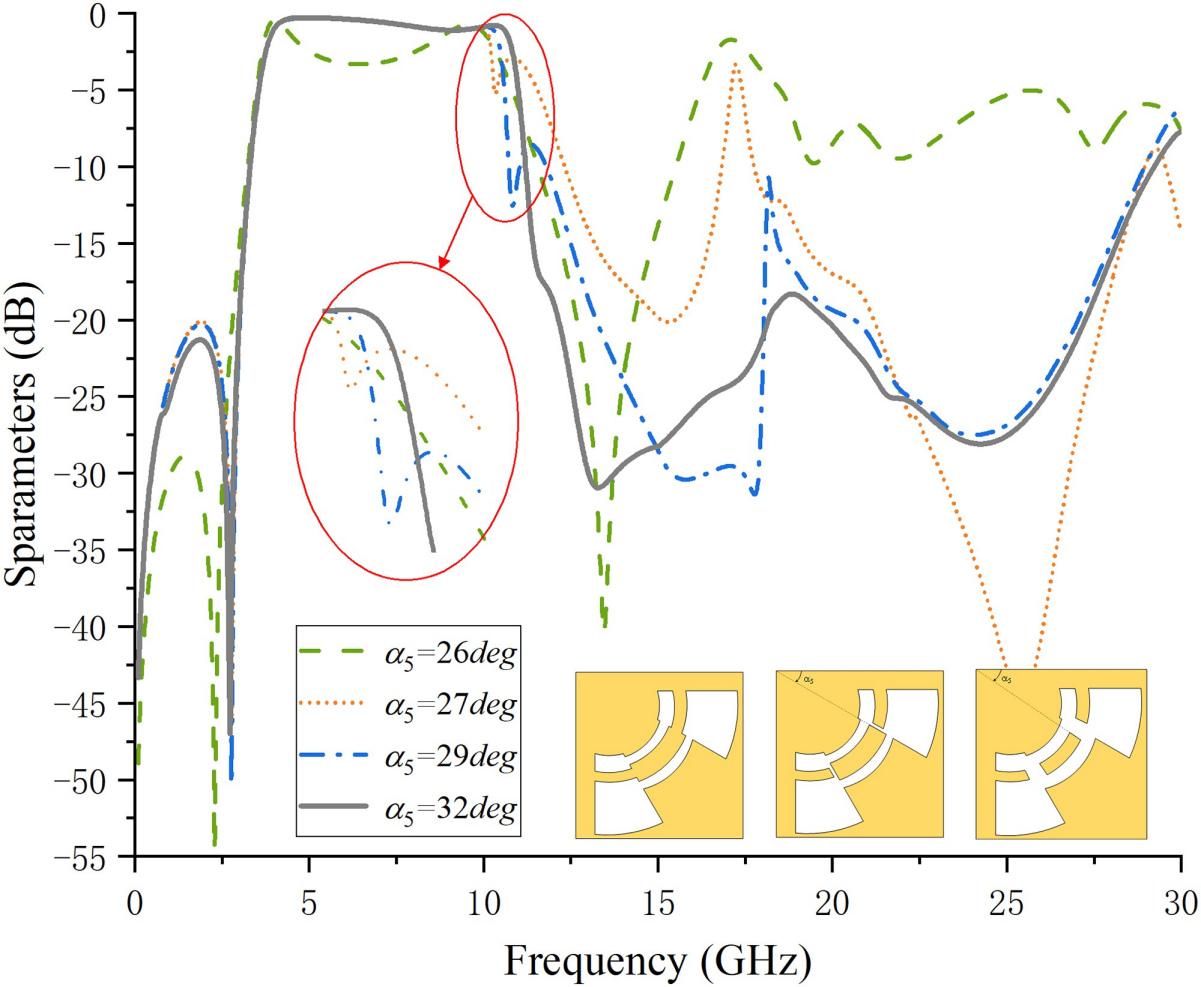
The corresponding S_21_ waveform when the DGS size increases.

Fig 9 shows the analysis of the influence of the DGS size on the passband under the premise that the ACPW determines the maximum bandwidth. *α*_5_ is the angle of introducing the DGS structure. When *α*_5_ = 26deg, DGS is exactly zero, which is the minimum critical value of DGS. Observing the waveform, it can be found that the S_21_ curve has the disadvantage of pass-band collapse when *α*_5_ = 26deg, and the stop-band characteristic is extremely poor. After analysis, the reason for the collapse of the passband may be the similar notch band caused by the coupling of the CPW_2_ center conductor of the ACPW with the lower side SIR resonator. Therefore, a DGS structure is introduced to reduce the coupling between the central conductor strip of CPW_2_ and the lower side SIR. It can be observed from the simulation results that with the increase of *α*_5_, the notch point in the passband gradually moves to the edge of the passband, the collapse bandwidth gradually decreases, and the resonance point outside the band also decreases continuously.

**Fig 10:**
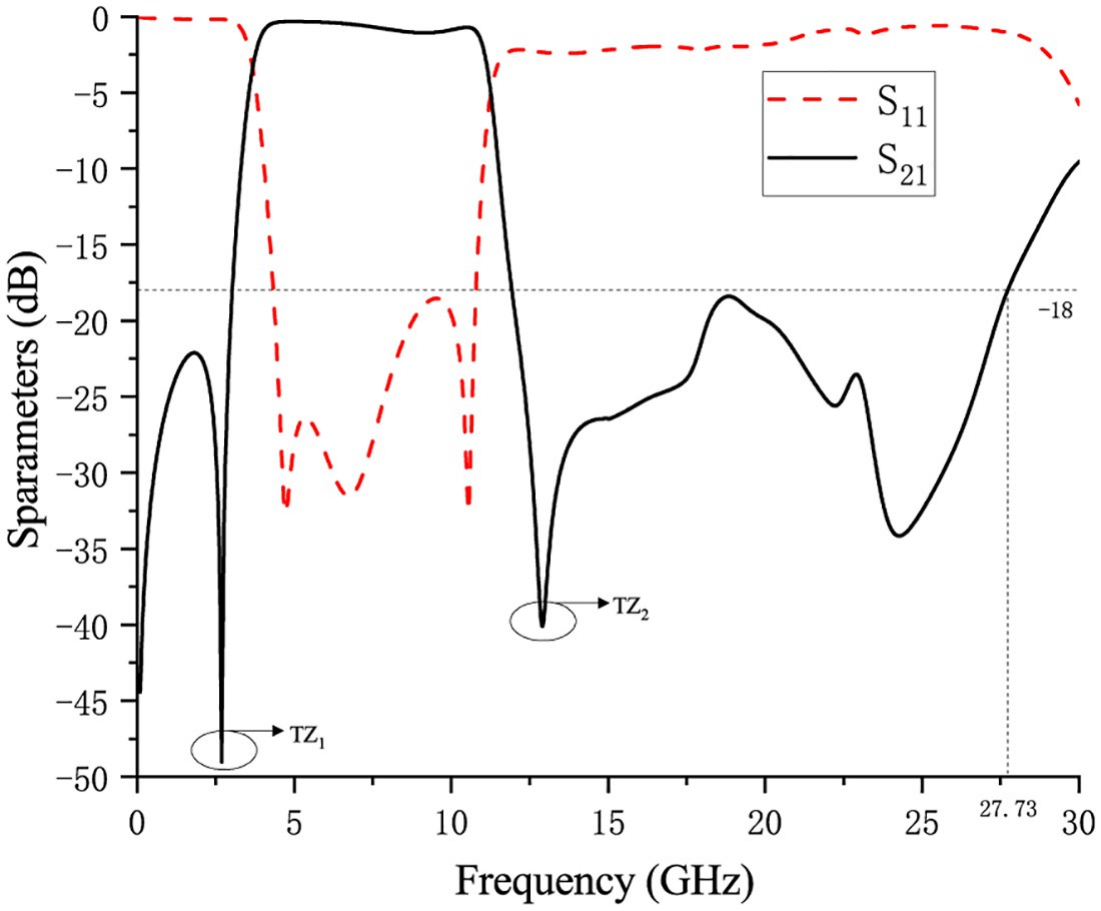
S_21_ simulation curve of UWB BPF.

Finally, after simulation debugging, it is determined that *α*_5_ = 34deg, and the S_21_ simulation curve at this time is shown in Fig 10. Ultra-wideband passband range is from 3.7 to 11.12GHz, insertion loss is less than 1dB, return loss is greater than 18dB, and the upper stopband is greater than 18dB until 27.72GHz, with two transmission zeros: TZ_1_ is located at 2.69GHz, and the attenuation reaches—49.04dB; TZ_2_ is located at 12.91GHz, and the attenuation reaches -40.10dB. Among them, the insertion loss near 9GHz is relatively large. On the one hand, the advantage of this is to ensure the attenuation depth of the transmission zero point, and on the other hand, it prevents the resonance from being distorted during the roll-off process. For the quasi-elliptic response filter [33], increasing its roll-off coefficient will lead to an increase in its in-band/out-of-band ripple, which will lead to an increase in the in-band insertion loss. In order to ensure good roll-off characteristics of the lower sideband, therefore sacrificing in-band insertion loss; in order to prevent distortion during the roll-off process, the second resonance point and the third resonance point should not be too close, which leads to an increase in insertion loss near 9GHz. In addition, the insertion loss near 9GHz also has certain advantages for the next step of notch introduction. While introducing the notch, the influence of the original insertion loss on the waveform can be ignored, and the depth of the notch can be further deepened.

### 2.4 UWB-BPF implementation

**Fig 11:**
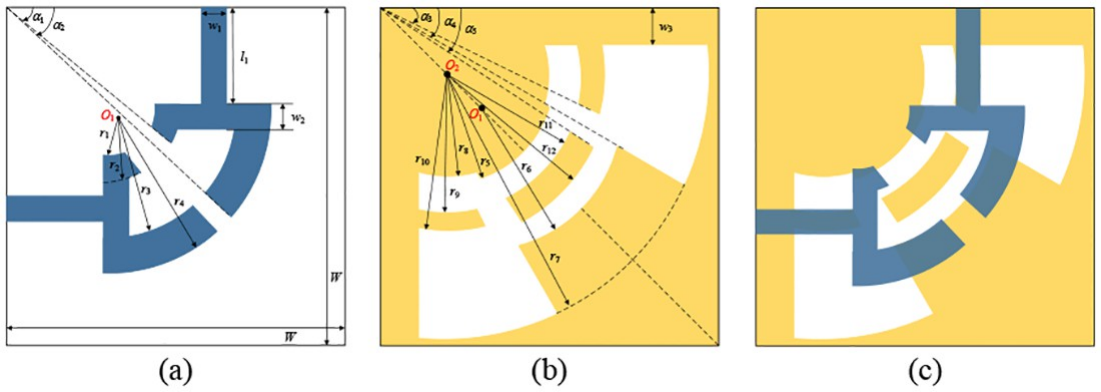
UWB BPF structure diagram. (a) the top layer. (b) the bottom layer. (c) UWB filter structure.

The UWB filter structure proposed in this paper is shown in Fig 11. A two-layer structure is introduced on the dielectric substrate, and the top layer is a microstrip circuit structure: it consists of a pair of back-to-back microstrip lines symmetrical with a 45° axis, and any section is composed of the input/output microstrip line and a vertical microstrip line connect two arcs with different tangent angles, as shown in Fig 11(a); the bottom layer is composed of ACPW-DGS structure, as shown in Fig 11(b). The UWB BPF is formed by vertical electromagnetic coupling between the top layer and the bottom layer, as shown in Fig 11(c). There is a vertical conversion transition through the part between the microstrip line and the coplanar waveguide. The enhanced capacitive coupling between the microstrip line and the coplanar waveguide can expand the position of the transmission zero point during this conversion process, so as to obtain a wider bandwidth [34]. In the part of the microstrip line and the coplanar waveguide coupling structure, in the case of high frequency, it can be equivalent to a parallel coupled line with a high degree of coupling, and the two transmission zeros generated are located at the low frequency end and one at the high frequency end [35]. At the same time, the microstrip line and the coplanar waveguide are facing each other, and the energy electromagnetic coupling between the two is very strong, so that good out-of-band suppression performance can be obtained [36]. After continuous optimization and testing, the final UWB BPF size is shown in Table 2. The coordinates of the center *O*_1_ are (3,3), and the coordinates of the center *O*_2_ are (2,2). Compared with other design methods, the hybrid microstrip/coplanar waveguide technology can make full use of the space of the top and bottom layers of the circuit board, which is conducive to the miniaturization of the circuit structure.

**Table 2:** Parameters of UWB filter structure.

symbol	value/mm	symbol	value/mm	symbol	value/deg
*w*_1_	0.8	*r*_5_	3.2	*α*_1_	35
*l*_1_	2.9	*r*_6_	4.1	*α*_2_	43
*w*_2_	0.9	*r*_7_	7.6	*α*_3_	26
*w*_3_	1.0	*r*_8_	3.0	*α*_4_	30
*r*_1_	1.3	*r*_9_	4.0	*α*_5_	34
*r*_2_	2.1	*r*_10_	4.6		
*r*_3_	3.8	*r*_11_	3.8		
*r*_4_	4.8	*r*_12_	4.5		

## 3 Dual notch UWB BPF design

**Fig 12:**
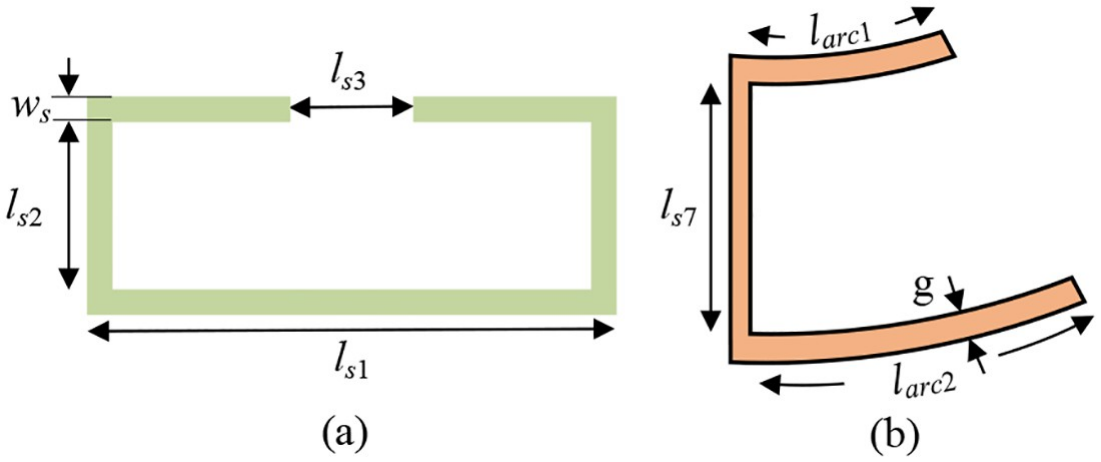
Notch structure diagram. (a) SRR. (b) CTR.

Placing notches in the UWB passband range can avoid interference caused by other passband systems. In this manuscript, on the basis of the UWB filter structure, a split ring resonator (SRR) and a pair of C-type resonators (CTR) are respectively coupled inside the ACPW structure of the backplane to place two notches, as shown in Fig 12(a) and 12(b) respectively. The center frequency of the notch is closely related to the length of the notch structure. By changing the length of the notch structure, the center frequency of the notch can be adjusted. Finally, the center frequencies are 9.17GHz and 9.96GHz by continuously adjusting the size of SRR and CTR. The physical lengths of the resonator structures are shown in Eqs (19) and (20), respectively:

$$\begin{array}{c} l_{\text{SRR}}=2l_{s1}-l_{s3}+2*w_{s}+4*l_{s2} \end{array}$$ (19)

$$\begin{array}{c} l_{\text{CTR}}=l_{arc1}+l_{arc2}+2*g+l_{s7} \end{array}$$ (20)

The notch frequency is related to the size of the resonator as follows in Eqs (21) and (22):

$$\begin{array}{c} f_{\left(9.17\text{GHz}\right)}\approx \frac{c}{2l_{\text{SRR}}\sqrt{\varepsilon_{\text{reff}}}} \end{array}$$ (21)

$$\begin{array}{c} f_{\left(9.96\text{GHz}\right)}\approx \frac{c}{2l_{\text{CTR}}\sqrt{\varepsilon_{\text{reff}}}} \end{array}$$ (22)

where *c* is the speed of light and *ε*_reff_ is the dielectric constant of the dielectric substrate.

**Fig 13:**
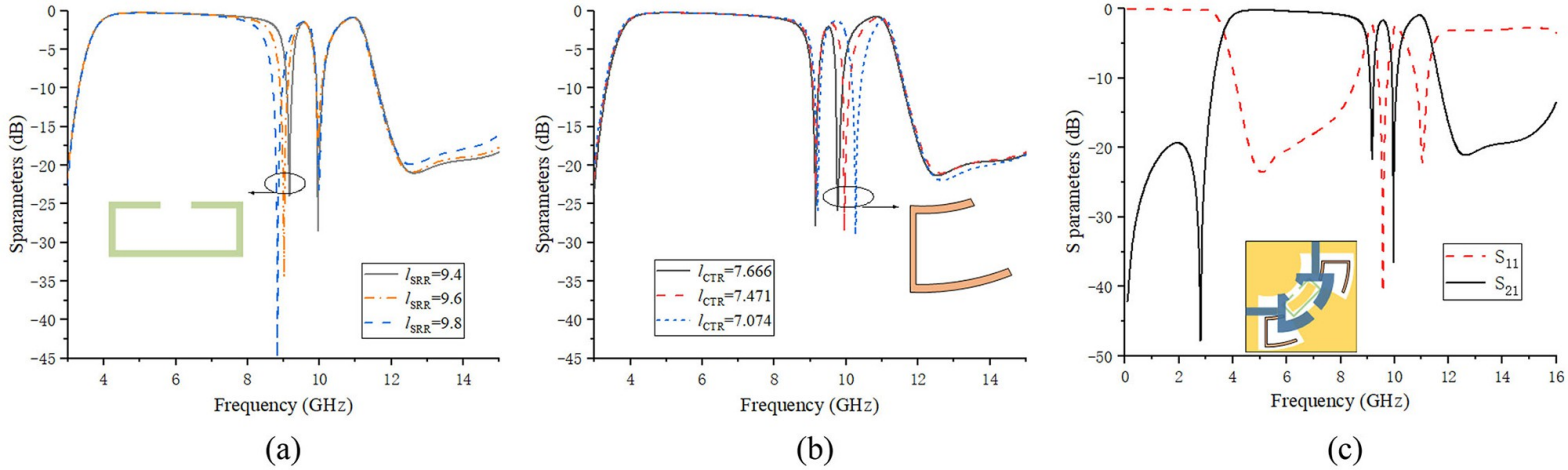
Notch characteristic analysis. (a) change the length of the SRR structure. (b) change the length of the CTR structure. (c) S_21_ waveform of the double-notch UWB BPF.

The center frequency of the notch can be adjusted by changing the physical length of the notch structure. The physical dimensions of SRR and CTR were changed respectively, and simulation verification was performed, and the corresponding S_21_ waveform change curves were obtained as shown in Fig 13(a) and 13(b) respectively. When the lengths of *l*_SRR_ are taken as 9.4mm, 9.6mm and 9.8mm respectively, it can be observed from Fig 13(a) that as the length of the first notch structure of the SRR increases, the center frequency of the first notch gradually moves to the low frequency direction, and the notch bandwidth keeps increasing. When the lengths of *l*_CTR_ are taken as 7.666mm, 7.471mm and 7.074mm, it can be observed from Fig 13(b) that with the decrease of the length of the second notch structure of CTR, the second notch center frequency gradually moves towards the higher frequencies. Finally, through continuous optimization and debugging, the center frequencies of the two notches are determined at 9.17GHz and 9.96GHz, and their notch depths are both greater than 20dB. The final S_21_ simulation waveform of the dual-notch UWB BPF is shown in Fig 13(c). At this time, the length parameters of the two notch structures are shown in Table 3.

**Table 3:** Parameters of two notch structures in millimetres (mm).

symbol	value	symbol	value
*l*_*s*1_	3.7	*l*_*arc*1_	1.8
*l*_*s*2_	1.2	*l*_*arc*2_	2.9
*l*_*s*3_	0.8	*w*_*s*_	0.2
*l*_*s*7_	2.2	*g*	0.2

**Fig 14:**
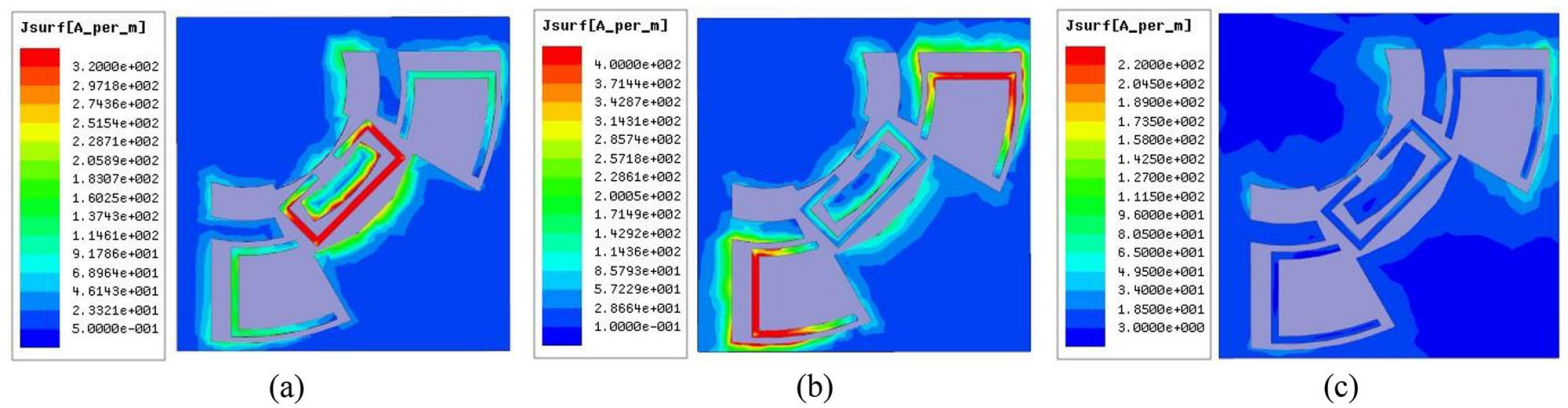
Surface current distribution diagram. (a) the center frequency is 9.17GHz. (b) the center frequency is 9.96GHz. (c) non-notch center frequency.

In order to further understand the influence of the notch structure on the UWB BPF, the current distribution on the filter surface is analyzed. Fig 14 shows the surface current distribution diagrams simulated at different notch center frequencies and non-notch center frequencies. When the simulated frequency is 9.17GHz, it can be seen from Fig 14(a) that the current is mainly concentrated on the SRR structure; when the simulation frequency is set to 9.96GHz, the current is mainly concentrated on the CTR structure, as shown in Fig 14(b); when the simulation frequency is at the non-notch center frequency, the obtained current distribution is shown in Fig 14(c), and the current is uniformly distributed throughout the BPF structure. By comparison, it is shown that the two structures of SRR and CTR are tightly coupled with ACPW, the structure corresponding to the introduction of the notches can be seen intuitively in the passband.

**Fig 15:**
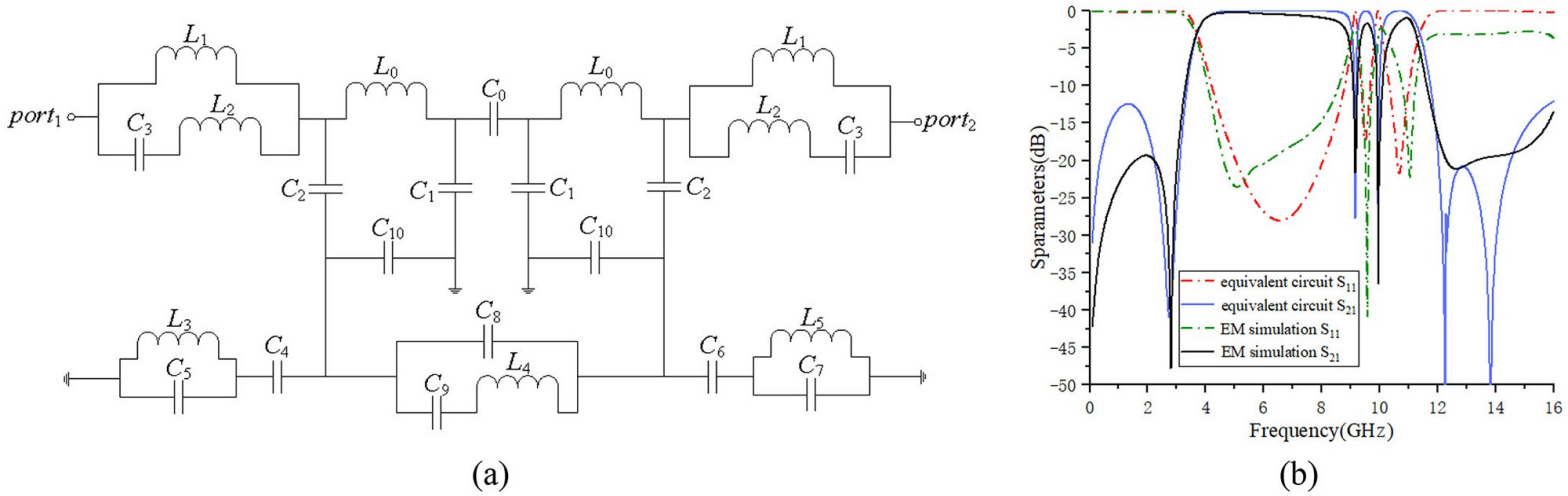
Equivalent circuit structure and comparison of simulation results. (a) lumped parameter equivalent circuit. (b) comparison of equivalent circuit and EM simulation results.

Fig 15(a) shows the approximate lumped-parameter equivalent circuit diagram of the designed UWB BPF with the dual notch. Fig 15(a) describes in detail the corresponding distribution relationship of lumped elements in the proposed model structure. Among them, the input/output feeder is equivalent to the loop formed by *L*_1_, *L*_2_, and *C*_3_, and the stub connected by the feeder is represented by *L*_0_ and *C*_1_ in parallel. *C*_1_ is mainly used to introduce the transmission zero point of the passband, and the two stubs are coupled to each other through *C*_0_. The vertical electromagnetic coupling between the two layers is mainly represented by the coupling of *C*_2_ and *C*_10_. The lumped parameters of the ACPW structure of the ground plane are equivalently composed of *C*_8_, *C*_9_, and *L*_4_. The two notch structures can be equivalent to a resonant circuit with *L* and *C* in parallel: the first notch structure SRR is equivalent to *L*_3_, *C*_5_ in parallel, the second notch structure CTR is equivalently connected in parallel by *L*_5_ and *C*_7_. The double-notch structure is coupled to the ACPW through *C*_4_ and *C*_6_. The position of the notch can be adjusted by optimizing the parameter values. The relationship between the double-notch position and its equivalent lumped parameter has a closely connected relationship, the specific relationship is given by Eqs (23) and (24).

$$\begin{array}{c} f_{\left(9.17\text{GHz}\right)}=\frac{1}{2\pi \sqrt{L_{3}\left(C_{4}+C_{5}\right)}} \end{array}$$ (23)

$$\begin{array}{c} f_{\left(9.96\text{GHz}\right)}=\frac{1}{2\pi \sqrt{L_{5}\left(C_{6}+C_{7}\right)}} \end{array}$$ (24)

The optimized lumped parameters are shown in Table 4. The comparison results of the response curve simulated by the equivalent circuit and the S-parameter curve simulated by HFSS are shown in Fig 15(b).

**Table 4:** Optimized lumped parameters.

symbol	value/nH	symbol	value/pF	symbol	value/pF
*L*_0_	3.3	*C*_0_	0.004	*C*_6_	0.06
*L*_1_	0.47	*C*_1_	1	*C*_7_	0.29
*L*_2_	1.06	*C*_2_	3	*C*_8_	0.26
*L*_3_	0.45	*C*_3_	0.11	*C*_9_	0.55
*L*_4_	0.75	*C*_4_	0.09	*C*_10_	0.24
*L*_5_	0.73	*C*_5_	0.58		

## 4 Dual-notch UWB design with upper stop-band characteristics

**Fig 16:**
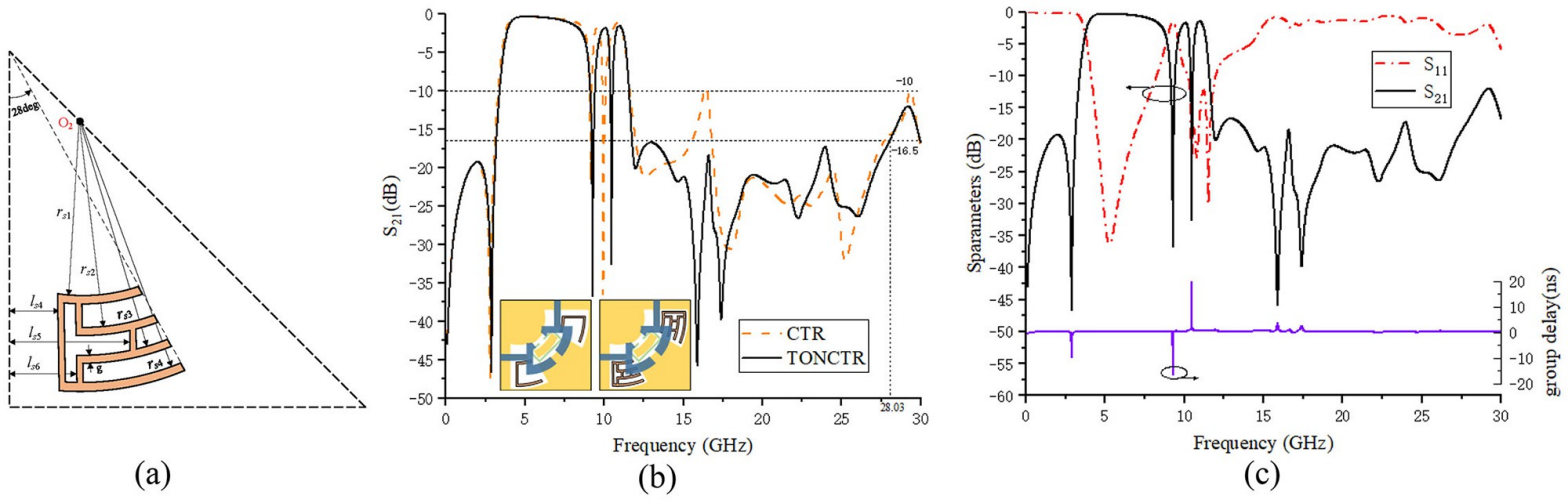
TONCTR structure and comparative response curve. (a) TONCTR structure diagram. (b) comparison of CTR and TONCTR response curves. (c) S-parameter simulation results and group delay results.

Above the second notch is introduced through the CTR, but the upper stop-band characteristics are not ideal. Therefore, on the basis of the CTR, it is improved by nesting the same type of resonator to form a new type called third order nested C-type resonator (TONCTR). The structure of TONCTR is shown in Fig 16(a), and its size parameters are shown in Table 5. By increasing the length of the resonator in this way, the purpose of reducing the frequency of the out-of-band resonance point is achieved, thereby increasing the out-of-band impedance. The S_21_ curve is obtained by simulation, and the comparison curve of the out-of-band response caused by the two resonators is shown in Fig 16(b).

**Table 5:** TONCTR dimensions in millimetres (mm).

symbol	value	symbol	value	symbol	value
*l*_*s*4_	1.2	*r*_*s*1_	4.8	*r*_*s*4_	7.2
*l*_*s*5_	3.2	*r*_*s*2_	5.6	g	0.2
*l*_*s*6_	1.7	*r*_*s*3_	6.4		

Finally, the S-parameter simulation results and group delay results of the dual-notch UWB BPF with good stopband characteristics are shown in Fig 16(c). Its UWB passband range is from 3.76 to 11.29GHz, the first passband is from 3.76 to 8.73GHz, and the insertion loss is 0.92dB; the second passband is 9.73–10.27GHz, and the insertion loss is 1.74dB; the third passband is 10.72–11.29GHz, the insertion loss is 1.46dB; its return loss is greater than 12.15dB. The center frequency of the first notch is 9.28GHz, and the notch depth reaches -36.77dB. The center frequency of the second notch is 10.48GHz, and the notch depth reaches -32.58dB. The influence of amateur radio band (9.2–10.3GHz) and X-band satellite link band (9.6–12.3GHz) on the UWB communication system is suppressed respectively. The passband has two transmission zeros, TZ_1_ is located at 2.89GHz, the attenuation reaches -46.62dB, and TZ_2_ is located at 12.02GHz, and the attenuation reaches -20.06dB. The upper stopband is greater than 16.5dB and lasts to 28.03GHz. There are three places where the group delay increases sharply, the first place corresponds to TZ_1_ respectively; the second and third places correspond to two notches respectively, and the rest of the passband is basically in a stable state.

## 5 Testing and verification

**Fig 17:**
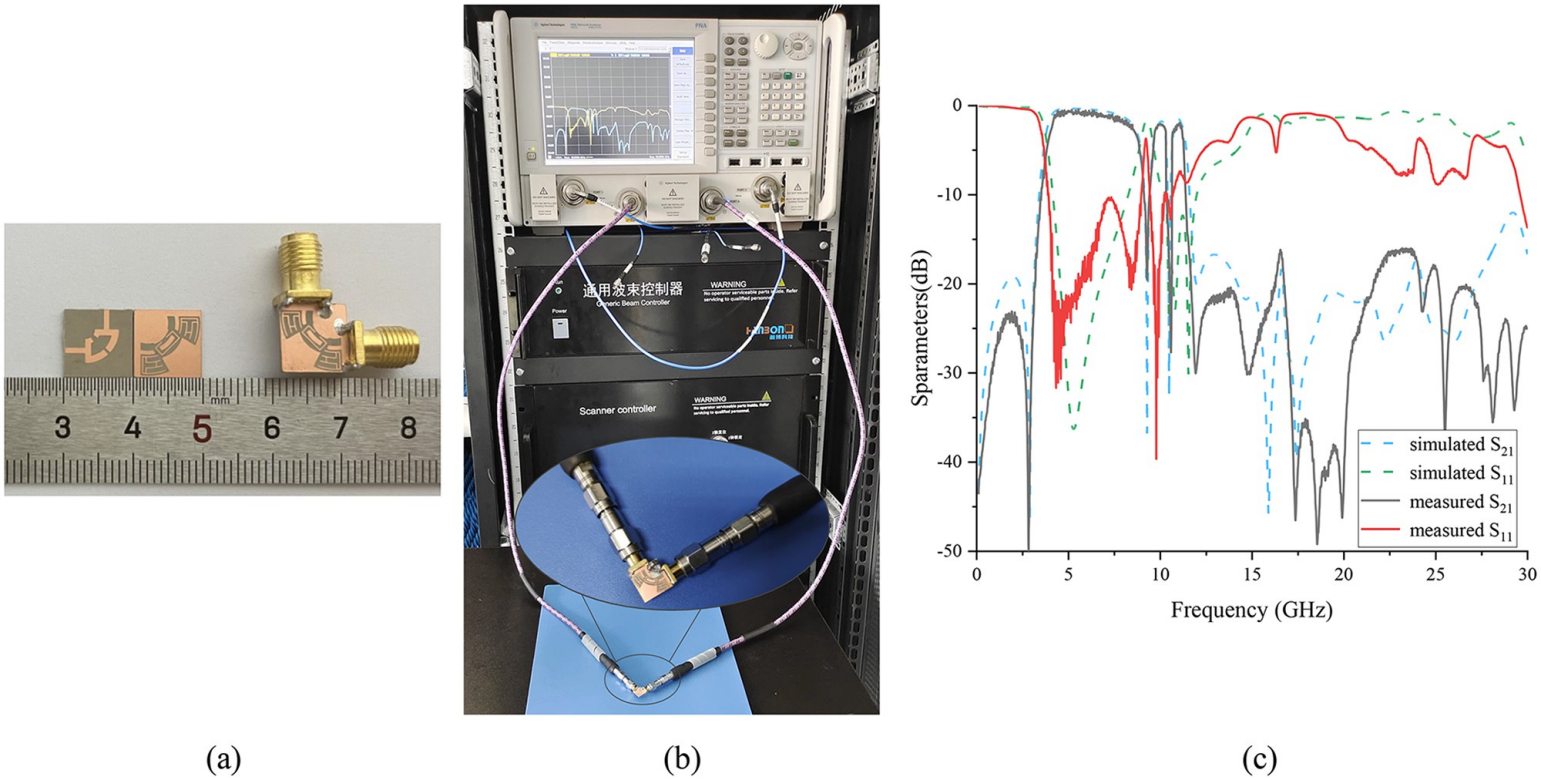
(a) the top and ground views of the fabricated prototype. (b) measurements are made using a vector network analyzer. (c) comparison of measured results and simulation results.

Compared with other filter designs, the filter has the following advantages in physical processing: no complicated processing technology is required, no drilling is required, and the dimensional accuracy of the board design is after one decimal point. Fig 17(a) shows the top and ground views of the fabricated prototype. The double-notch dielectric substrate adopts Rogers RT/duroid 6006, its dielectric constant is 6.15, the loss tangent is 0.0019, the thickness of the substrate is 0.635 mm, and the size is only 9.8 mm × 9.8 mm. Use the vector network analyzer Agilent N5247A to test the developed prototype, as shown in Fig 17(b). And compare the test results with the simulation results, as shown in Fig 17(c). The measured results and the simulation results have the same trend of change, with good consistency, but there are some deviations between the two. The cause of the error may be the influence of machining error, test environment, and simulation accuracy.

The UWB filter designed in this manuscript is compared with the [3, 5, 33–44], and the results are shown in Table 6. As can be seen from the second column of Table 6, the filter in this paper is different from others in structure, it adopts the improved ACPW-DGS. It can be seen from the third column that the structure proposed has transmission zeros in both the upper and lower passbands unlike the [5, 37, 42, 44], which has only one transmission zero, whereas [38, 41, 43] do not possess transmission zero. Columns 4, 5, and 6 describe that the structure in this design has a relative bandwidth of 3dB of 110%, the return loss is greater than 12.15dB, and there are two notches at 9.28GHz and 10.48GHz, all of which are greater than 20dB. Different from other narrow stop-band filters [5, 37, 40, 43, 44], the filter proposed has good wide stop-band characteristics with attenuation greater than 16dB, lasting up to 30GHz. Compared with other references, the filter designed in this manuscript is compact and has the smallest electrical Dimensions.

**Table 6:** Comparison of filter parameters between this paper and other filters.

Ref.	The main structure	*f*_L_ *f*_H_ at TZs	FBW(%)	NBs / attenuation	S.B. rejection	Size (λ_*g*_×λ_*g*_)
[37]	MSLLR	×,✔	120	NA	-18dB to 17GHz	0.55×0.51
[38]	*	×,×	104.3		NA	0.25×0.16
[39]	COS+MMR	✔,✔	110.2		NA	0.39×0.11
[40]	R/L-HTL	✔,✔	110.9		-18dB to 30GHz	0.22×0.20
**UWB filter with notch characteristic**						
[3]	CPW to Microstrip	✔,✔	113	6,8.05/>18	-17dB to 16GHz	0.99×0.73
[5]	LPF+HPF+DGS+DMS	×,✔	135.5	5.8/>60	-26dB to 15GHz	0.47×0.26
[33]	CPW to Microstrip	✔,✔	137	5.5,7.9/>17	-26dB to 15GHz	0.26×0.14
[34]	CPW to Microstrip	✔,✔	112	5.28,8/>19	-23dB to 16GHz	0.44×0.33
[35]	CPW to Microstrip	✔,✔	111	4.55,8.07/>15.5	-19dB to 16GHz	0.99×0.73
[36]	CPW to Microstrip	✔,✔	116	6.1,8.1/>15.5	-15dB to 18GHz	0.44×0.33
[41]	SISLR+DMS	×,×	102.2	6.2,8/>10	NA	0.57×0.23
[42]	U-DGS+SIR	×,✔	110	5.6,6.7/>20	NA	0.96×0.96
[43]	PDMS+EMMR	×,×	106.6	5.3,5.9,6.4,7.4/>15	-10dB to 17GHz	0.61×0.34
[44]	CPW to Microstrip	×,✔	116	5.3,6.25,8.28/>13	-17dB to 15GHz	1.08×0.67
this	ACPW-DGS	✔,✔	110	9.28,10.48/>20	-16dB to 30GHz	0.20×0.20

Ref.: Reference; *: DGS+Interdigitated+SIR+Shunt Stub; *f*_L_ *f*_H_ at TZs: Transmission zeros at lower and upper bandpass edges; ×: TZ absent;✔: TZ exists; FBW: fractional bandwidth; NBs: notch bands; S.B.: Stopband; NA: not available; λ_*g*_: the guided wavelength at 6.85GHz.

## 6 Conclusion

In this manuscript, the traditional ground plane CPW structure is optimized and evolved, proposed a new structure ACPW-DGS, which is combined with the top plane microstrip line to form UWB-BPF through vertical electromagnetic coupling. The proposed structure has two transmission zeros at the edge of the passband, which can enhance the selectivity of the passband and provide a good passband response; there are two notches in the passband to avoid interference; transmission zeros outside the passband are available to enhance the stop-band characteristics. In addition, the design of the filter is compact and the size is small, which meets the design requirements for the miniaturization of the filter.

## Supporting information

S1 Data Comparison of measured results and simulation results. (CSV)
